# Small ocean temperature increases elicit stage-dependent changes in DNA methylation and gene expression in a fish, the European sea bass

**DOI:** 10.1038/s41598-017-10861-6

**Published:** 2017-09-29

**Authors:** Dafni Anastasiadi, Noelia Díaz, Francesc Piferrer

**Affiliations:** 1 Institute of Marine Sciences (ICM-CSIC), Passeig Marítim, 37–49, 08003 Barcelona, Spain; 2 Present Address: Max Planck Institute for Molecular Biomedicine, Regulatory Genomics Lab, Röntgenstraße 20, 48149 Münster, Germany

## Abstract

In natural fish populations, temperature increases can result in shifts in important phenotypic traits. DNA methylation is an epigenetic mechanism mediating phenotypic changes. However, whether temperature increases of the magnitude predicted by the latest global warming models can affect DNA methylation is unknown. Here, we exposed European sea bass to moderate temperature increases in different periods within the first two months of age. We show that increases of even 2 °C in larvae significantly changed global DNA methylation and the expression of ecologically-relevant genes related to DNA methylation, stress response, muscle and organ formation, while 4 °C had no effect on juveniles. Furthermore, DNA methylation changes were more marked in larvae previously acclimated to a different temperature. The expression of most genes was also affected by temperature in the larvae but not in juveniles. In conclusion, this work constitutes the first study of DNA methylation in fish showing that temperature increases of the magnitude predicted by the latest global warming models result in stage-dependent alterations in global DNA methylation and gene expression levels. This study, therefore, provides insights on the possible consequences of climate change in fish mediated by genome-wide epigenetic modifications.

## Introduction

In natural fish populations, elevated temperatures associated with global warming^1^ can result in shifts in a variety of phenotypic traits, mostly related to reproduction and life history^2^. However, how environmental cues are perceived and integrated is still poorly understood and constitutes a central topic in ecological developmental biology^3,4^. In recent years, evidence is accumulating on the impact of environmental factors on epigenetic mechanisms^5,6^ which directly regulate gene expression and lead to lasting phenotypic consequences^7,8^. It is also becoming more evident that epigenetic changes contribute to the observed phenotypic plasticity^9,10^, and thus it has been argued the importance of incorporating epigenetics into ecological research^11^.

In aquatic poikilotherms, temperature has effects on global DNA methylation regardless of the phylum considered. In a marine invertebrate, the Antarctic polychaete (*Spiophanes tcherniai*), the response to an increase in temperature of 5.5 °C was accompanied by an increase of DNA methylation at specific CpGs^12^. In fish, environmental temperature constitutively influences global DNA methylation, since species living in different latitudes have different amounts of 5-methylcytosine^13^. Nevertheless, the effects of temperature on epigenetic mechanisms have been only studied in a few species. In Atlantic cod (*Gadus morhua*), an increase of 4 °C during incubation resulted in changes in the expression of genes involved in one carbon and DNA methylation pathways^14^. In half-smooth tongue sole (*Cynoglossus semilaevis*) exposed to a 6 °C temperature elevation, up to ~73% of genotypic females underwent sex-reversal. Furthermore, ~94% of the offspring of the heat-treated fish developed as males even in the absence of elevated temperature, suggesting transmission of the altered state^15^. The testis of these sex-reversed females at high temperature showed a ~10% increase of DNA methylation at the genome-wide level when compared to the ovaries of unaffected females, but had similar levels to the testes of normal males^15^. In Nile tilapia (*Oreochromis niloticus*), a genome-wide increase of DNA methylation was observed with a water temperature increase of 8 °C^16^. However, it remains to be established whether temperature increases of the magnitude predicted by the latest global warming scenarios (i.e., in the range of 1–4 °C^1,17^) can affect global DNA methylation. This is particularly relevant if the exposure takes place during early development when fish seem to be more sensitive^18–21^, with potential long-lasting phenotypic consequences^20,22^.

The European sea bass (*Dicentrarchus labrax*) is a marine fish distributed across the North Atlantic, the Mediterranean and the Black Sea^23^. It inhabits coastal waters to a depth of 100 m as well as brackish waters in estuarine areas and coastal lagoons^24,25^. Adult sea bass typically live in temperatures from 8°–24 °C but spawning and early development takes only place in the range of 13–17 °C^25–27^. Importantly, as for the majority of marine teleosts^28^, sea bass spawns pelagic eggs which float in the upper layers, where the potential effects of climate change can be more pronounced^29,30^. Previous studies have shown that sea bass is sensitive to temperature changes, in particular during early development, from 0 to around 60 days fertilization (dpf), which is considered its thermosensitive period (TSP), affecting a variety of morphological and physiological aspects^31–34^. One of these aspects is population sex ratio, which was shown to be mediated, at least in part, by DNA methylation changes in a key gene involved in sex differentiation^35^.

The goal of this study was to determine whether small temperature variations experienced during different periods of fish early life, and within the range established by the latest global warming models, can affect global DNA methylation and the expression of a suite of ecologically important genes relevant for survival and development. We focused on two sub-periods within the TSP^31,32,34^. We show that the first 15 days of life are critical since changes in temperature that include this period can elicit changes in both DNA methylation and gene expression.

## Materials and Methods

### Animals and general rearing conditions

The broodstock that produced the eggs and larvae used in this study was reared following the Moretti *et al*.^36^ and Díaz *et al*.^37^ protocols. Briefly, European sea bass broodstock originating from an unselected population with low genetic variation (Φ_ST_ = 0.008) was reared in 5000-liter tanks at the aquarium facilities of the Institute of Marine Sciences under natural conditions of temperature and photoperiod, corresponding to 41°23′6″ N latitude, with pH ~7.9, salinity ~37.8 ppt and oxygen saturation at 85-100%. Animals were fed a commercial pellet food (EFICO, BioMar) of the appropriate size as described in detail in Díaz *et al*.^37^.

European sea bass females produce floating pelagic eggs of ~1 mm diameter that are fertilized upon release. Fertilized eggs batches were collected between December and April following natural spawnings with the aid of a surface overflow collector system. Eggs were gently rinsed in seawater, measured in a 1-liter glass graduate cylinder and the floating portion, containing the viable eggs, was divided in equal parts and placed in between three and five 19-liter PVC cylindrical containers, depending on batch size. Each 19-liter container, provided with its own water supply and aeration, and fitted with a bottom nylon mesh, was randomly assigned to one of two 650-liter fiberglass tanks (mesocosm) thermoregulated at 14 ± 0.5 °C, which coincided with the temperature of the broodstock tanks during the spawning season. The 650-liter mesocosms were completely covered to maintain eggs in the darkness. At 14 °C hatching usually takes place ~3 days post fertilization (dpf). Once the yolk sac was reabsorbed larvae were fed with Artemia AF (INVE Aquaculture, Belgium), followed by enriched Artemia EG (INVE Aquaculture, Belgium) and progressively switched to natural conditions of photoperiod. Juveniles were fed *ad libitum* with pelleted food (ProAqua S.A. Spain) according to their size and adapted to their thermal regimes. (See ethics statement at the end of this section).

### Temperature treatments

This study involved two different experiments carried out within the TSP, from ~0 to ~60 dpf^31,32,34^. For a summary of the experimental design and temperature treatments, see Fig. S1 and Table S1.

#### Experiment 1

Fish were exposed to elevated temperature as eggs and larvae and this experiment consisted of two parts. In experiment 1.1, three groups were reared at a constant temperature of either 15 °C, considered the control temperature for European sea bass eggs and young larvae^31,32^, 17 °C (+2 °C) or 19 °C (+4 °C), from 0 to 15 dpf. Within a group, temperature fluctuations were always <1 °C. Experiment 1.2 involved a total of six groups: three groups were acclimated at 15 °C since fertilization and then changed to 19 °C at either 15 hours post fertilization (hpf); group 15–19(15), 120 hpf; group 15–19(120) or 240 hpf; group 15–19(240). The other three groups were acclimated first to 19 °C and then changed to 15 °C at either 15 hpf; group 19-15(15), 120 hpf; group 19-15(120) or 240 hpf; group 19-15(240). A detailed explanation of the procedure for achieving the desired temperatures can be found in the Additional Supporting Information and Fig. S2. All groups were sampled at 15 dpf when they were still larvae. The whole experiment 1 was repeated 5 times with different egg batches each time.

#### Experiment 2

In this experiment, fish were reared under a naturally-fluctuating temperature, starting at 14 °C when they were eggs and reaching 17 °C by the time they reached 20 dpf. Then, fish were randomly split into two groups and placed in two replicated 650-liter mesocosms. One group was reared at 17 °C while in the other the temperature was progressively increased at 0.5 °C/day until it reached 21 °C. The exposure to elevated temperature included the 20-60 dpf period. At 60 dpf all fish were sacrificed. The 21 °C temperature, in addition to represent a 4 °C increase with respect to 17 °C was also chosen because of its known effects on the sexual phenotype^35^. Each treatment was carried out in duplicate. Since sampling took place at 60 dpf, we refer to the sampled fish as juveniles in order to clearly distinguish them from the fish from experiment 1, which were sampled at 15 dpf when they were still larvae.

### Sampling

Fish were not fed the day before sampling. Fish were immediately frozen in liquid nitrogen and stored at −80 °C. Due to the small size of larvae and juveniles the whole fish was taken in order to get, from the same individual, enough DNA for assessing global DNA methylation and enough RNA for determining gene expression. Thus, all available fish were sacrificed at the moment of sampling. Standard length and body weight were determined at the time of sampling in each experiment. Differences in length and weight were tested with the Kruskal-Wallis rank sum test for experiment 1, and with the Wilcoxon rank sum test for experiment 2.

### Methylation Sensitive Amplified Polymorphism (MSAP)

Methylation-Sensitive Amplified Polymorphism (MSAP) was used to determine the effects of different water temperatures and water temperature changes on global DNA methylation. For experiments 1.1 and 1.2 with larvae, 10–17 fish were pooled together and considered as one biological replicate, while for experiment 2 with juveniles one biological replicate corresponded to one individual fish. Biological replicates from each group were as follows for larvae: 15 °C, n = 23; 17 °C, n = 15; 19 °C, n = 12; 15–19(15), n = 16; 15–19(120), n = 15; 15–19(240), n = 12; 19-15(15), n = 14; 19-15(120), n = 15; 19-15(240), n = 12; and for juveniles: 17 °C, n = 20, and 21 °C, n = 20 samples. The MSAP method was a modification of the protocol used by Morán and Pérez-Figueroa^9^, in turn based on Reyna-López *et al*.^38^ and Xu *et al*.^39^. The restriction enzymes used were *Msp*I and *Eco*RI or *Hpa*II and *Eco*RI. A detailed description of the protocol, including DNA extractions, is presented in the Additional Supporting Information and an overview of raw MSAP data in Fig. S3.

### MSAP: raw data processing and classification of fragments

The details of raw data processing, including the definition of amplicon size categories and how the genotyping error per primer was calculated can be found in Additional Supporting Information. The scoring of electropherograms was performed using the package RawGeno (v. 2.0.2)^40^ in R (v. 3.2.5) and Rstudio (v. 1.0.136)^41,42^. Further MSAP analyses were performed using the R package *msap* (v. 1.1.9)^43^ following a band-based strategy^44^. The bands were scored as unmethylated (Type I), methylated in the inner cytosine (Type II), hemi-methylated in the outer cytosine (Type III) or hypermethylated (Type IV). Then, they were classified as methylation-susceptible (MSL) or non-methylated (NML) and transformed into binary matrices indicating methylated/unmethylated MSL according to *msap* ^43^. Three rounds of binning and scoring were performed: (1) 15 °C vs. 17 °C vs. 19 °C (0-15 dpf), (2) 15 °C vs. 15–19 °C vs. 19-15 °C (0-15 dpf) and (3) 17 °C vs. 21 °C (20-60 dpf). Further details can be found in Additional Supporting Information and the numbers of loci in Table S2.

### MSAP: estimation of differentiation of DNA methylation

The binary matrix of polymorphic MSL was used in order to estimate differentiation of DNA methylation between groups by Analysis of Molecular Variance (AMOVA)^45^ with 1,000,000 permutations. The Φ_ST_ indicates the level of differentiation of DNA methylation between the groups being compared. Differentiation of DNA methylation between groups was visualized by a Principal Coordinates Analysis (PCoA)^46^.

Significant single loci were identified after adjusting by the method of Benjamini and Hochberg (FDR < 0.05) the *p*-values obtained from multiple Fisher’s exact tests for count data on the polymorphic MSL^47^. In the case of temperature acclimation (experiment 1.2), the common significant loci between the two types of temperature changes (15 °C to 19 °C and 19 °C to 15 °C) were identified. A pairwise dissimilarity matrix was calculated based on Gower’s distances of categorical variables and used to perform hierarchical clustering by the Unweighted Pair Group Method with Arithmetic Mean (UPGMA).

### Quantitative real-time PCR (qRT-PCR)

In experiments 1.1 and 1.2 with larvae, total RNA was extracted from 4 pools per group, containing ~10 whole larvae each pool. In experiment 2 with juveniles, RNA was extracted from the upper body trunk of 4 individual juveniles per group. The expression of the following European sea bass genes was measured (only exons were targeted, as based on the European sea bass genome annotation^48^): the DNA-methyltransferases 1 and 3 (*dnmt1* and *dnmt3*, respectively; the latter orthologous to *dnmt3b1* of *Danio rerio*), heat shock protein 70 (*hsp70*), glucocorticoid receptor (*nr3c1*), insulin-like growth factor-1 (*igf1*), myogenin (*myog*), long melanopsin (*opn4α*), thyroid hormone receptor*-α* (*tr-α*), and trypsinogen 2 (*tryp2*). The elongation factor-1 alpha (*ef-1a*) and the 40 S ribosomal protein S30 (*fau*) were used as reference genes and had been previously validated in sea bass^49^. Further details on RNA extractions and qRT-PCR are fully described in the Additional Supporting Information.

### qRT-PCR data analysis

Quantitative RT-qPCR data were collected by the SDS 2.4 software (Applied Biosystems) and Cq values were calculated using the RQ Manager 1.2.1 (Applied Biosystems). Measured Cq values were corrected for primer efficiency Cq_E_ = Cq(log_(1+E)_/log_2_)^50^ and further analyses were performed using the R packages ReadqPCR (version 1.16.0) and NormqPCR (version 1.16.0)^51^. The geometric mean of the two reference genes was used to normalize the expression of target genes. Relative gene expression was calculated using the 2^−ΔΔCq^ method and errors were estimated using standard propagation of error method^52^. For all statistical analyses, dCq values were used to compare each group against the 15 °C group for experiment 1 and 17 °C for experiment 2, always within the same developmental stage.

The Shapiro-Wilk normality test was used to check if data conformed to a normal distribution, while the homoscedasticity of variances was checked by the Levene’s test. Then, the means were compared by the Student’s two-tailed *t*-test for pairwise comparisons. False discovery rate (FDR) was controlled by estimating the *q*-values^53^ of multiple two-group comparisons presented together.

### Data availability

All data generated and analysed during this study are included as Supplementary Information files. These include the binary files used as inputs for msap, those with the methylation-sensitive polymorphic loci (outputs of msap) on which the statistics were based and the dCq values of RT-qPCR.

### Ethics statement

Our facilities are approved for animal experimentation by the Ministry of Agriculture and Fisheries (certificate number 08039–46–A) in accordance with the Spanish law (R.D. 223 of March 1988). Fish were treated according to the European Convention for the Protection of Vertebrate Animals used for Experimental and Other Scientific Purposes (ETS Nu 123, 01/01/91). The experimental protocol was approved by the Spanish National Research Council (CSIC) Ethics Committee within the project AGL2013–41047–R. Fish were sacrificed using an overdose of 2-phenoxyethanol (2PE).

## Results

### Global DNA methylation

**Figure 1:**
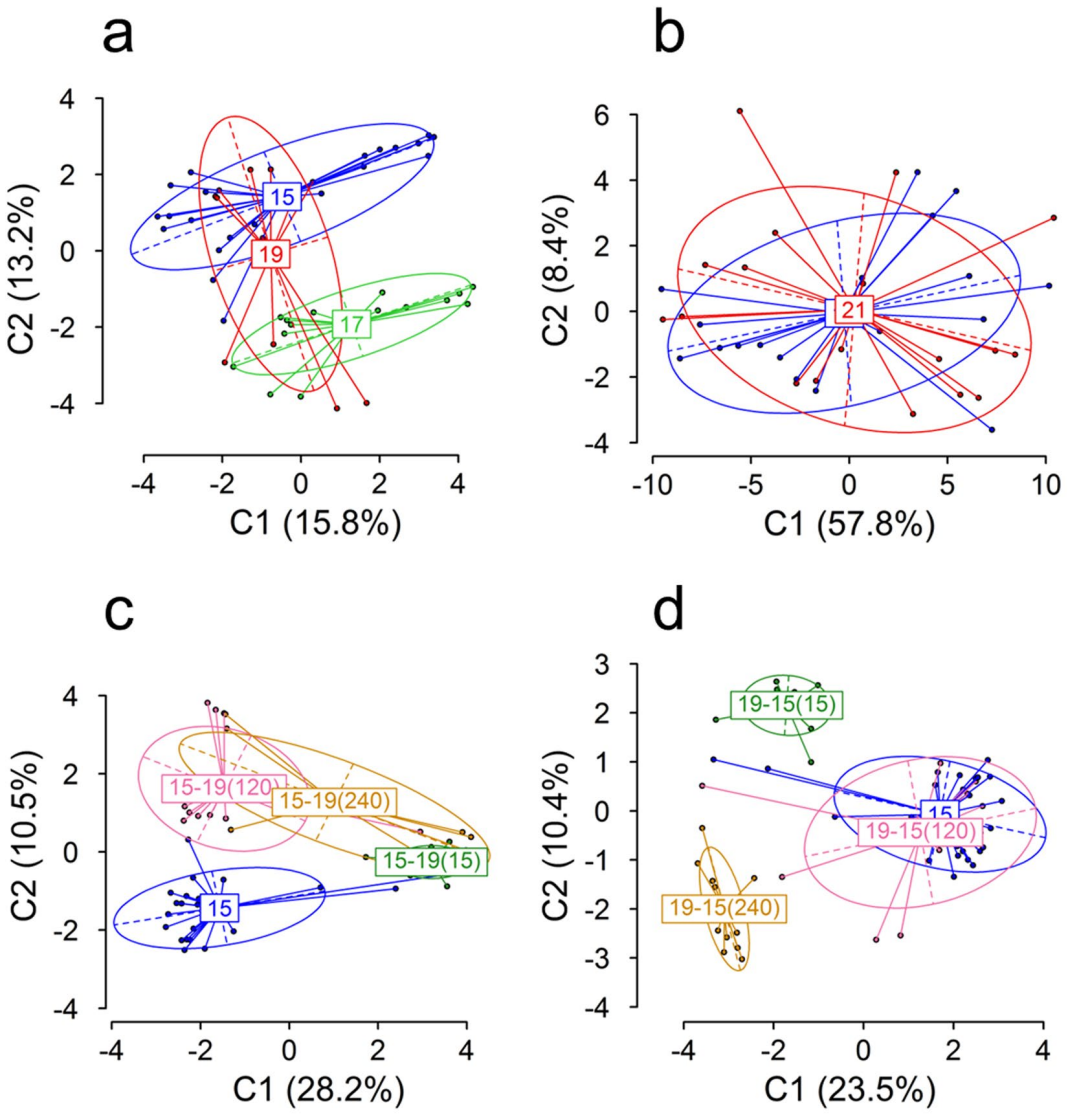
Global DNA methylation in European sea bass larvae (**a**, **c**, **d**) and juveniles (**b**) subjected to constant temperatures (**a**, **b**) or acclimated at different stages during development (**c**, **d**) and visualized by Principal Coordinates Analysis (PCoA). (**a**) Larvae reared at 15 °C [blue, n = 21], 17 °C [green, n = 15] or 19 °C [red, n = 12]. (**b**) Juveniles reared at 17 °C [blue, n = 18] or 21 °C [red, n = 18]. (**c**) Larvae switched to 19 °C at 15 [15–19(15), green, n = 15], 120 [15–1915–19(120), pink, n = 15] and 240 [15–19(240), yellow, n = 10] hours post fertilization. (**d**) Larvae switched to 15 °C at 15 [19-15(15), green, n = 14], 120 [19-15(120), pink, n = 15] and 240 [19-15(240), yellow, n = 11] hours post fertilization. The two first components of the PCoA are shown. Values in parenthesis indicate the percentage of the variance explained. Group labels indicate the centroid, the ellipses show the group dispersion, the dotted lines the direction of maximum and minimum dispersion (long and short axis of the ellipse, respectively) and the points represent individual samples from the different groups.

In experiment 1.1, determination of differentiation of DNA methylation between groups by Analysis of Molecular Variance (AMOVA) based on the distance matrix calculated from binary polymorphic MSL showed significant differences (*p* < 0.001) in global DNA methylation between larvae that had been kept at a constant temperature of 15, 17 or 19 °C since day 0 (experiment 1.1), with a Φ_ST_ of 0.1626 among groups (Table 1; see Table S3 for pairwise comparisons). Differentiation of DNA methylation between groups was visualized by a Principal Coordinates Analysis (PCoA; Fig. 1a). In contrast, in experiment 2 AMOVA found non-significant differences (*p* = 0.42) in global DNA methylation in 60 dpf juveniles that had been exposed to a constant temperature of 21 °C between 20 and 60 dpf when compared to siblings reared at a constant temperature of 17 °C (Table 1; Fig. 1b). Regarding the six groups of larvae that were exposed to temperature changes after a period of acclimation (experiment 1.2), AMOVA showed that there were also significant differences (*p* < 0.001) in DNA methylation of fish acclimated from 15 °C to 19 °C at 15, 120 or 240 hpf, as well as in DNA methylation of fish acclimated from 19 °C to 15 °C at 15, 120 or 240 hpf. Furthermore, the Φ_ST_ value was approximately twice as high (Φ_ST_ ~0.357) with respect to the groups of experiment 1 (Φ_ST_ = 0.1626) exposed to different but constant temperatures (Fig. 1c,d; Table 1). Thus, differences of 2 °C affected DNA methylation in larvae but differences of 4 °C did not affect DNA methylation in juveniles.

**Table 1:** AMOVA results of MSAP based on polymorphic methylation-susceptible loci (MSL).

Period (dpf)	Temperature	Treatment (°C)		d.f.	SSD	MSD	Variance	Φ_ST_	*p*
0–15	Constant	15 *vs*. 17 *vs*. 19	Among groups	2	238.6	119.360	5.761	0.1626	0
			Within groups	45	1335.0	29.670	29.670		
			Total	47	1574.0	33.480			
	Acclimation	15 → 19	Among groups	3	449.5	149.8	8.793	0.3611	0
			Within groups	59	916.7	15.54	15.54		
			Total	62	1366	22.04			
	Acclimation	19 → 15	Among groups	3	462.3	154.1	8.987	0.3531	0
			Within groups	59	970.4	16.45	16.45		
			Total	62	1433	23.11			
20–60	Constant	17 *vs*. 21	Among groups	1	27.6	27.580	−0.032	−0.00115	0.4168736
			Within groups	34	957.6	28.170	28.170		
			Total	35	985.2	28.150			

Abbreviations: d.f., degrees of freedom; dpf, days post fertilization; MSD, mean square deviations; MSL, methylation-susceptible loci; SSD, sums of square deviations.

**Figure 2:**
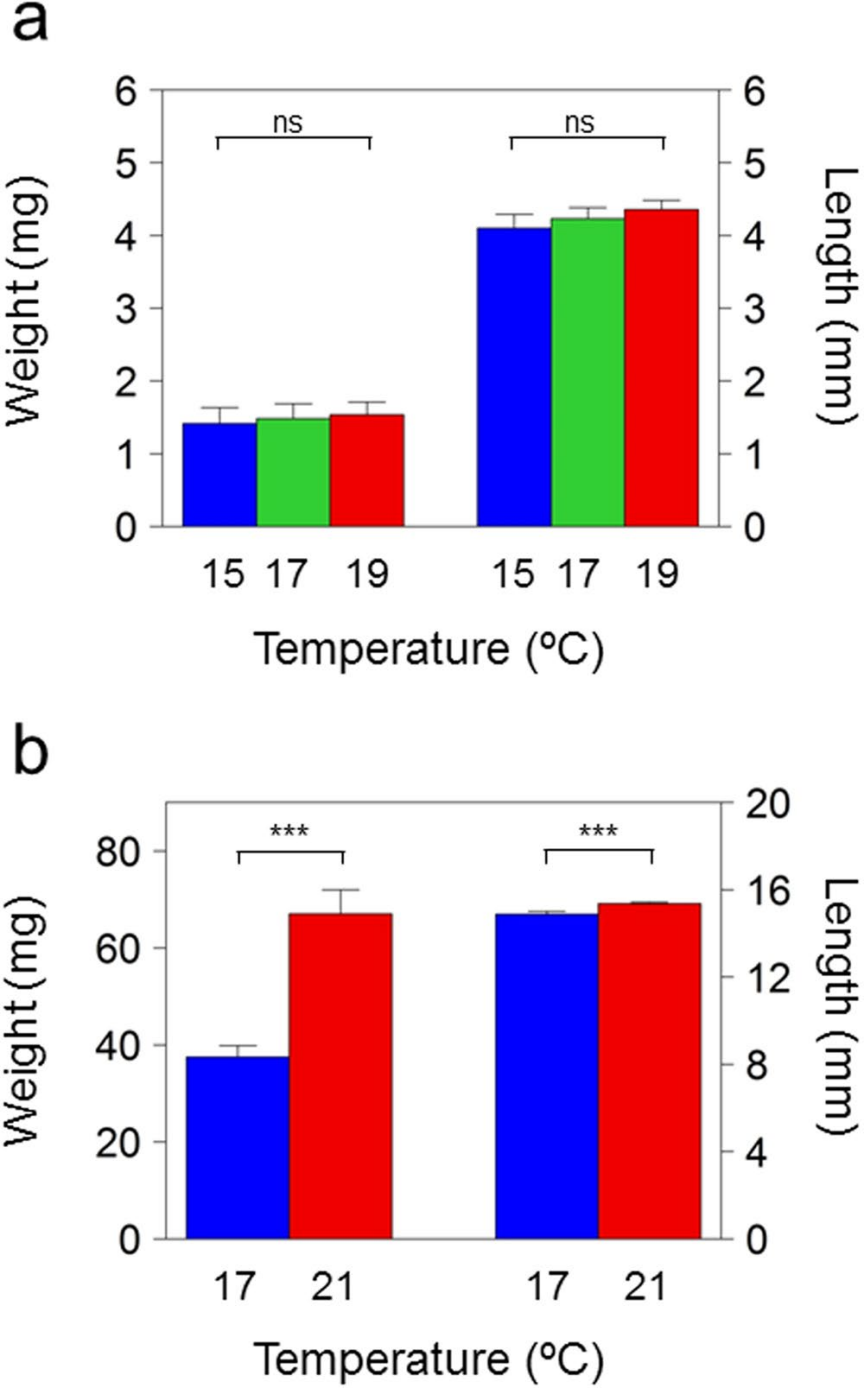
Weight and standard length of sea bass reared at constant temperatures during the thermosensitive period of development. (**a**) Larvae of 15 days post fertilization (dpf) reared at 15 °C (n = 10), 17 °C (n = 10) and 19 °C (n = 10). (**b**) Juveniles of 60 dpf reared at 17 °C (n = 20) or 21 °C (n = 20) from 20 to 60 dpf. Statistical differences were calculated by the Kruskal-Wallis rank sum test for the 0–15 dpf period and the Wilcoxon rank sum test for the 20–60 dpf period and are shown for each stage with the following equivalence: ***p ≤ 0.001, ns = not significant.

Growth was determined at the end of each treatment period in the groups of fish subjected to different but constant temperatures to assess possible effects of growth on DNA methylation. No significant differences were found between larvae of the 15, 17 and 19 °C groups after 15 days of temperature treatment (weight: Kruskal-Wallis χ^2^ = 0.15946, *p* = 0.9234; length: Kruskal-Wallis χ^2^ = 1.5785, *p* = 0.4542) Fig. 2a. However, juveniles reared at 21 °C had a significantly higher weight (Wilcoxon rank sum test [W] = 317, *p* = 0.001117) and length (W = 374, *p* = 0.000002) compared to juveniles reared at 17 °C, indicating that the 40 days (20-60 dpf) at a constant 21 °C had stimulated growth compared to 17 °C (Fig. 2b).

To further explore the effect of temperature on global DNA methylation differences, the 15 and 19 °C groups (experiment 1) and the 17 and 21 °C (experiment 2) groups were taken and the MSAP bands were grouped into the following categories: unmethylated (Type I), internal cytosine methylation (Type II), hemi-methylated (Type III) and full methylation (Type IV)^43,54,55^. In addition, global methylation was calculated as the ratio: Types II + III/scorable informative loci (Types I + II + III)^55–57^. There was an increasing tendency of Type I bands in the 19 °C larvae and 21 °C juveniles, of Type III bands in the 19 °C larvae and a decreasing tendency of the ratio in the 19 °C larvae and 21 °C juveniles (Fig. S4).

**Figure 3:**
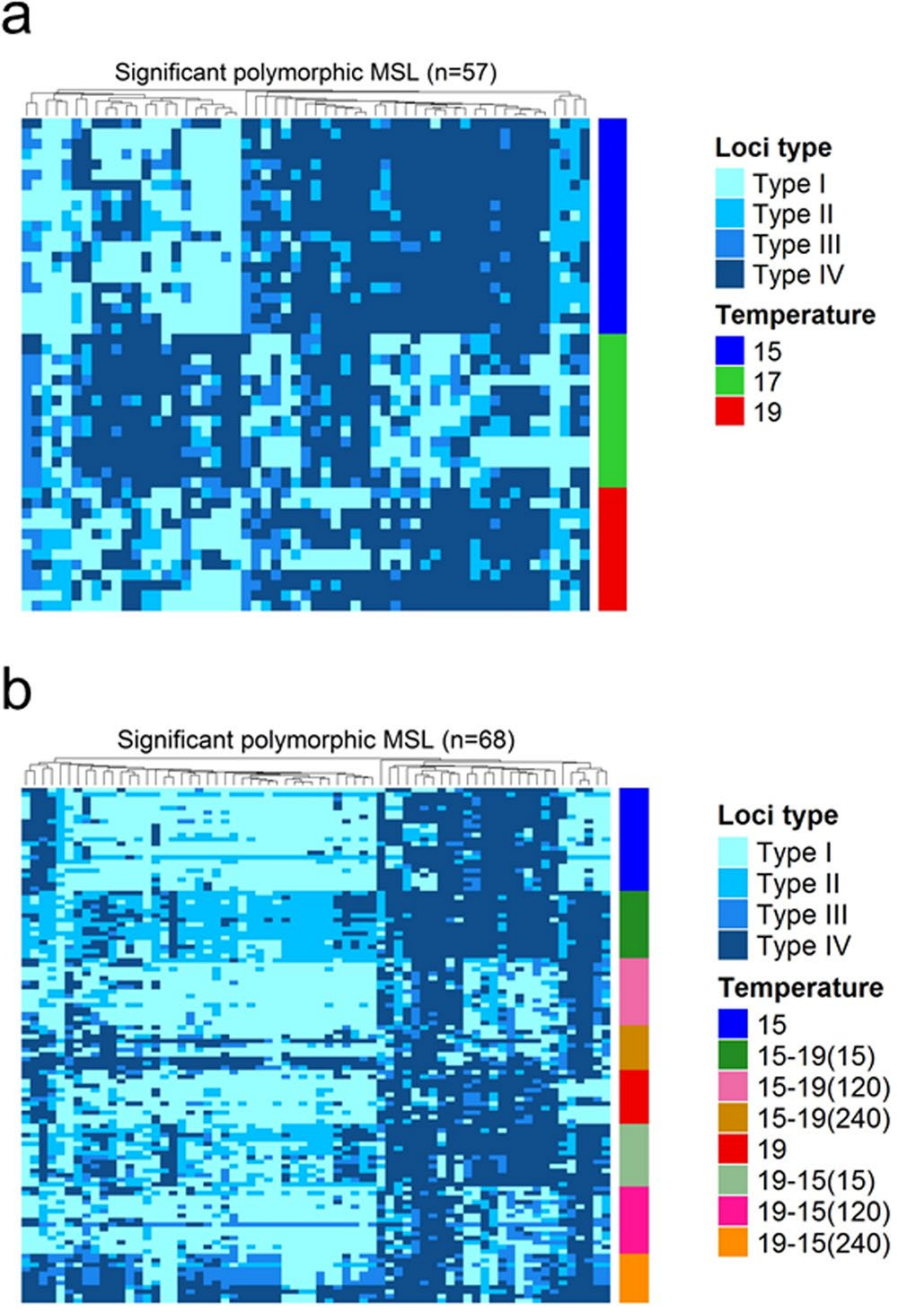
Significant polymorphic methylation susceptible loci (MSL) in larvae. (**a**) Significant loci between groups of larvae subjected at 15 °C, 17 °C or 19 °C from 0 to 15 days post fertilization plotted as a heatmap (n = 57 significant loci). (**b**) Significant loci between groups of larvae subjected to constant 15 °C or 19 °C, and larvae switched from 15 °C to 19 °C at 15 hours post fertilization (hpf; 15–19(15)), 120 hpf (15–19(120)) or 240 hpf (15–19(240)), or switched from 19 °C to 15 °C at 15 hpf (19-15(15)), 120 hpf (19-15(120)) or 240 hpf (19-15(240)). In this case, the n = 68 significant loci common to both types of temperature switches, (15 °C to 19 °C and 19 °C to 15 °C) were identified and used for the heatmap. Significant single loci were identified after applying multiple Fisher’s exact tests and adjusting the obtained *p*-values by the method of Benjamini and Hochberg. Only loci with an FDR < 0.05 were used in the analysis. A hierarchical clustering using the Unweighted Pair Group Method with Arithmetic Mean (UPGMA) based on pairwise Gower’s distances of categorical variables was applied for the loci. Four types of loci were used: Type I, unmethylated; Type II, inner cytosine methylation; Type III, hemi-methylation of the outer cytosine; Type IV, full methylation.

The significant loci based on Fisher’s exact tests (FDR < 0.05) were 57/298 (19.1%) polymorphic MSL for the 15 °C vs. 17 °C vs. 19 °C (0-15 dpf) comparison (Fig. 3a) and 0/216 (0%) for the 17 °C vs. 21 °C (20-60 dpf) comparison. Between the groups acclimated from low to high temperature there were 97/231 (42.0%) significant polymorphic MSL and between the groups acclimated from high to low temperature there were 109/204 (53.4%) significant polymorphic MSL detected. Between the two comparisons, there were 68 significant polymorphic MSL in common (Fig. 3b).

### Gene expression during development

**Figure 4:**
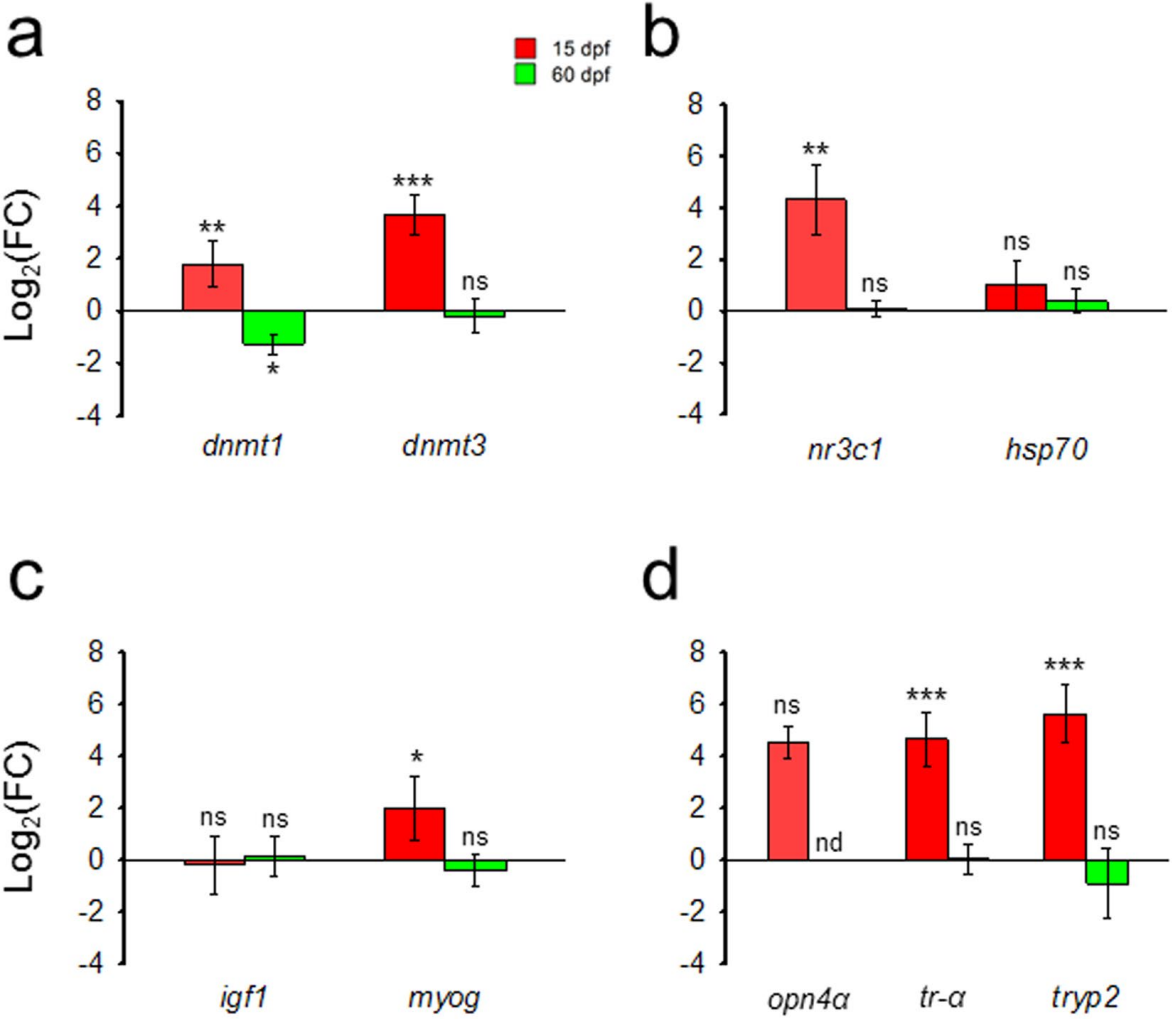
Gene expression changes in fish exposed to high *vs*. low temperature sampled when larvae at 15 days post fertilization (dpf) or when juveniles at 60 dpf. (**a**) Genes related to DNA methylation: DNA (cytosine-5-)-methyltransferase 1 (*dnmt1*) and DNA (cytosine-5-)-methyltransferase 3 (*dnmt3*); (**b**) Genes related to stress: glucocorticoid receptor (*nr3c1*) and heat shock cognate 70 protein (*hsp70*); (**c**) Genes related to muscle growth and differentiation: insulin-like growth factor 1 (*igf1*) and myogenin (*myog*). (**d**) Genes related to organ/tissue formation/function: long melanopsin (*opn4α*), related to visual system; thyroid hormone receptor alpha (*tr-α*), related to metamorphosis; and trypsinogen 2 (*tryp2*), related to the digestive system. Data are shown as log_2_-transformed fold change (log_2_FC) values and error bars indicate the confidence intervals. For each gene, log_2_FC values were calculated comparing the +4 °C group with respect to their corresponding low temperature at either 15 (19 °C vs 15 °C) or 60 dpf (21 °C vs 17 °C), which was always set at 0 in the log scale (n = 4). For each gene, statistical differences were calculated by pairwise Student’s two-tailed *t*-tests and corrected for multiple comparisons and are shown between each group and its corresponding control temperature group (asterisks), with the following equivalence: *q < 0.05, **q < 0.01, ***q < 0.001, ns = not significant, nd = not determined.

To determine possible effects of temperature other than in global DNA methylation, the expression of several genes was measured in the +4 °C groups and compared to the expression of the control groups within each treatment period (experiment 1.1 with larvae: 0-15 dpf; experiment 2 with juveniles: 20-60 dpf). In general, increased temperatures had profound effects on gene expression in larvae but not in juveniles, where in most cases significant differences were not observed. Thus, in larvae exposed to constant elevated temperature, expression levels of the maintenance (*dnmt1*; Student’s *t-*test [t] = 4.6662, *q* = 0.0049) and the *de novo* (*dnmt3*; t = 7.2864, *q* = 0.0008) DNA-methyltransferases were significantly increased. In contrast, in juveniles elevated temperatures significantly (t = −3.4465, *q* = 0.0265) decreased *dnmt1* while it had no effect on *dnmt3* (t = −0.1438, *q* = 0.7250; Fig. 4a). With regards to genes involved in stress and heat shock response, *nr3c1* and *hsp70*, respectively, temperature significantly (t = 5.1679, *q* = 0.0042) upregulated *nr3c1* expression in larvae but not in juveniles (t = 0.2550, *q* = 0.7250), while *hsp70* was not affected (larvae: t = 1.8523, *q* = 0.1371; juveniles: t = 1.1556, *q* = 0.3525; Fig. 4b). Regarding the expression of genes involved in muscle differentiation and growth, *igf1* and *myog*, elevated temperature increased only *myog* in larvae (larvae: t = 2.8813, *q* = 0.0380; juveniles: t = −0.9127, *q* = 0.4509), while *igf1* was not affected (larvae: t = −0.3090, *q* = 0.7250; juveniles: t = 0.1201, *q* = 0.7250; Fig. 4c). Finally, regarding the genes involved in organ development, temperature increased the expression of *tr-α* (t = 7.3106, *q* = 0.0008) and *tryp2* (t = 8.427, *q* = 0.0008) in larvae but not in juveniles (*tr-α*: t = −0.0815, *q* = 0.7250; *tryp2*: t = −0.5582, *q* = 0.6088; Fig. 4d), while there was no effect on *opn4α* (larvae: t = 8.3686, *q* = 0.00551 and juveniles: not detected; Fig. 4d).

## Discussion

In this study we provide evidence that elevated temperatures during the initial larval stages of the European sea bass result in changes in DNA methylation and gene expression. To assess DNA methylation, we opted for MSAP because it allows determining global DNA methylation and is cost-effective when dealing with a large number of samples, as in our case. MSAP is typically and widely used in ecologically-oriented studies^10,11,57–62^ to query a high number of samples and to explore global effects. With this technique, downstream higher resolution can be achieved by Sanger sequencing of polymorphic bands identified by gel electrophoresis. However, we chose to automatize fragment analysis to avoid the introduction of errors by manual scoring of gel electrophoresis, a potential risk of this approach. MSAP was applied taking into account all necessary quality controls and accepted standards. We use the term global DNA methylation in line with the literature^60,61,63,64^, since it allows to draw conclusions on global DNA methylation differentiation, and not at a single nucleotide resolution. The MSAP experimental procedure and fragment analysis used in this study produced a number of bands which represents a significant portion of the theoretical maximum number of bands predicted by *in silico* digestion of the sea bass genome. In particular, the number of polymorphic methylation-sensitive loci (MSL) available for analysis was in the uppermost range of the number of analyzed MSL in similar ecologically-oriented studies using the same technique^59,64,65^. Lastly, the results on hemi-methylated DNA observed in this study are comparable to what has been observed using MSAP in other fish species^47,66^, as well as in other vertebrates^59,67^.

In the present study, we show that even small temperature increases of just 2 °C resulted in significant changes in global DNA methylation if experienced during larval development, but not if experienced only during the larval-to-juvenile transition of sea bass. Interestingly, thermal acclimation in the range of 4 °C during the first 15 days of life elicited changes in DNA methylation, both at the global level and at the number of significant single loci. These changes were of a nearly double magnitude than the changes detected between fish subjected to different but constant temperature during the same period. Thus, larval stages are particularly sensitive to even small temperature increases, and, further still, more sensitive when the change in temperature takes place in animals previously acclimated to a different (lower or higher) temperature, which could be associated with the developmental stage of acclimation. These findings are in accordance with the developmental sensitivity windows as defined by the interactions of time, dose and phenotypic modifications^21^. In this study, we minimized the confounding effect of genetic variation on DNA methylation^68^ by using full-sibs, while the only distinct environmental factor was temperature. In addition, temperature had no effect on the growth of larvae, while there was an effect in juveniles. Together, this reinforces that the observed differences in global DNA methylation in larvae but not in juveniles are due to a direct effect of temperature and not as a consequence of differences in genetics or growth rates affected by different temperatures. Due to the small fish size we were only able to use pools of whole larvae and parts of the juvenile fish which were necessarily a mixture of cell types concealing the cell-specific DNA methylation patterns. Thus, we are not able to exclude changes in cell types and population numbers contributing to the observed changes in DNA methylation in larvae *vs* juveniles.

Despite the change in global DNA methylation observed due to temperature in groups of larvae subjected to different temperatures, there was no obvious effect on the type of MSL fragments. This could be due to changes from one methylation status to another and *vice versa*, e.g. from a hemi-methylated to an unmethylated status. This has been established in different fish tissues where temperature can both hyper-^15,16,34^ and hypo-methylate^16,69^ gene-specific CpG sites. Furthermore, temperature can persistently affect the methylation status of individual CpG sites in sea bass^34^ and other fish species^15^. Thus, the effects observed in experiment 1 would most likely remain as permanent effects as fish grow. Moreover, during fertilization and early embryogenesis in mammals, genome-wide reprogramming events occur that erase and reestablish the epigenetic marks^70^. In fish, the reprogramming events during early development take place shortly after fertilization and are completed during embryogenesis^71–73^. Indeed, temperature increases of 7.5 °C during this sensitive period affected the expression of DNA-methyltransferases in zebrafish (*Danio rerio*), although no global changes in DNA methylation were evident^74^. These reprogramming events, however, occur well before the time fish were sampled in this study, and hence do not interfere with the observations made with 15 dpf larvae and 60 dpf juveniles. Together, our results contribute to the definition of the window of sensitivity to environmentally induced persistent epigenetic modifications^75^ in an ecologically relevant context.

As a proxy for phenotypic consequences of temperature changes we used gene expression levels. We selected a subset of genes essential for survival and development; related to DNA methylation, stress response and tissue and organ formation. Most of the studied genes showed a clear stage-dependent response to different constant temperatures. The DNA-methyltransferases (*dnmt1*, *dnmt3*) and the myogenin (*myog*) were affected by temperature, as observed for other fish species^14,69,74,76–80^. The increase in the expression of DNA-methyltransferases should be linked with the differences in DNA methylation of the 15 °C vs. 19 °C larvae. Thyroid hormone receptor alpha (*tr-α*) and trypsinogen 2 (*tryp2*) are temporally regulated during fish development^81–84^. Interestingly, the anti-freeze glycoprotein present in Notothenoid fishes is derived from a trypsinogen-like protein^85^ and consequently there is extensive literature on the role of trypsin and trypsinogen in the molecular basis of cold adaption in Antarctic fish^86^ including those exposed to warming episodes^87^. Here it is interesting to note that of all the genes analyzed in our study the one that had the highest fold-induction was precisely *tryp2*. A similar trend was found for the expression of glucocorticoid receptor (*nr3c1*), which is permanently upregulated by temperature increase in fish^88,89^. Thus, this permanent upregulation of *nr3c1* gene expression by temperature could be traced to the earliest developmental stages. In contrast, the expression of heat shock cognate protein 70 (*hsp70*) was not affected by temperature in accordance with its constitutive nature^90^. The lack of effects on *hsp70* found in our study is also consistent with what has been observed in acclimation studies in other fish^91^. Similarly, there was a lack of temperature effects on the insulin-like growth factor (*igf1*), in accordance with the absence of long-lasting changes in its expression status with temperature^89^. A transcriptomic response related to changes of the overall metabolism at high temperature^92,93^ could influence the dynamic regulation of gene expression, although in this study gene expression was normalized using two stable reference genes. Consistent with the effects of temperature on global DNA methylation depending on the timing of the occurrence, there was a general increase of gene expression with constant temperature increase and stage-dependent influences in the larvae, but no effect in the juveniles.

It is widely accepted that DNA methylation is one of the most important epigenetic mechanisms regulating gene expression^7,94^ and therefore the phenotype. In this study, we observed significant changes in global DNA methylation, and gene expression as a function of temperature. However, we cannot conclude that there was a causal relationship between DNA methylation and gene expression due to the approaches used although, of course, such relationship is likely to occur, as we have shown previously with a targeted gene approach involving gonadal aromatase (*cyp19a1a*) and functional studies^34^. Furthermore, temperature can also affect other epigenetic mechanisms, such as histone modifications^95^ or changes in the abundance of specific non-coding RNAs^96^, also contributing to the observed changes in the regulation of gene expression^97^.

In the context of global warming, epigenetic mechanisms are viewed as of increasing relevance for the survival of species^98^ since epigenetically-mediated phenotypic plasticity may be maladaptive or may serve as a buffer in rapidly changing environments^99–101^. It has been argued that research in ecological epigenetics must include not only field studies but also controlled studies with non-model organisms^62^. The temperature changes fish experienced in this study are ecologically relevant. The most vulnerable stages of sea bass life include from eggs to pre-metamorphosis larvae. In the European sea bass, early development following spawning occurs at 13–17 °C although most often at 13–15 °C. The current best estimates of warming in the upper 100 m of major ocean basins are 0.6–2.0 °C over the next 100 years^1^, although in coastal waters like the ones the European sea bass lives it can be up to 4 °C^17^. The temperature increases of 2–4 °C used here represent, therefore, the upper scenario within the scope of the increase of temperatures predicted by the latest global change models. Since MSAP is ideally suited for analyzing a large number of samples at a very reasonable cost, samplings could be extended to different natural populations of the same species living under different temperatures to determine whether similar effects as the ones reported here are found.

In summary, this study shows for the first time in fish that constant moderately elevated temperatures or temperature changes within the range predicted by the latest global warming models result in differences in global DNA methylation. Importantly, these effects are stage-dependent, since they are evident in larvae but not in juveniles. However, effects in juveniles cannot be excluded with longer times of exposure or higher temperatures. We also found that these global DNA methylation changes were accompanied by changes in the expression of ecologically important genes relevant for survival, development and organ formation. Thus, future studies on the possible consequences of climate change in fish should take into account the effects at the level of the epigenetic mechanisms regulating gene expression and, while sampling in the field, the existence of sensitive developmental windows.

## Electronic supplementary material

Supporting Information

Supporting Information

Supporting Information

Supporting Information
