# Supplementary material for: Small ocean temperature increases elicit stage-dependent changes in DNA methylation and gene expression in a fish, the European sea bass
Source: Sci Rep. 2017 Sep 29;7:12401. doi: 10.1038/s41598-017-10861-6 (PMC5622125; doi:10.1038/s41598-017-10861-6)
Supplement: Supplementary file 1 — Supporting Information [file 41598_2017_10861_MOESM1_ESM.pdf]

Small ocean temperature increases elicit stage-dependent changes in DNA methylation and gene expression in a fish, the European sea bass

Dafni Anastasiadi, Noelia Díaz, Francesc Piferrer

Institute of Marine Sciences (ICM-CSIC), Barcelona, Spain

Supplementary Online Information

Supplementary Material and Methods

Further details on temperature treatments of experiment 1

To achieve the desired temperature, and in order to avoid inducing a thermal shock, the nylon mesh bottom of the 19-liter PVC cylindrical container was blocked to prevent water loss and the container was gently moved to the next +1°C or -1°C mesocosm, depending on the polarity of change, until the desired temperature was achieved. These floating containers had an individual water supply to maintain water renewal at 30% $vol{\cdot}h^{-1}$ and two individual stone air diffusers to maintain dissolved oxygen saturation levels ~85-90%. The 650-liter tank had in turn its own air and water supplies (saturation levels ~85-100%). Thus, there were a total of twelve 650-liter mesocosms, the two at 14±0.5°C, which always received the fertilized eggs, and 10 additional mesocosms for the temperature experiments: two at 15±0.5°C, two at 16±0.5°C, two at 17±0.5°C, two at 18±0.5°C and two at 19±0.5°C (Fig. S2). Temperature changes were performed at steps of 1°C/2 h. In this way, groups passed through different 650-liter mesocosms. This procedure was repeated until the final designed temperature was reached (Fig. S2). Also, the tank effect was avoided since the 19-liter PVC cylindrical container passed through all the 650-liter mesocosms with the different temperatures (except for the 17°C group, which just passed through half of the 650-liter mesocosms).

DNA extractions

DNA was extracted from pools of larvae (experiment 1) and individually from lower body trunk of juveniles (experiment 2). The samples were immersed in digestion buffer (0.1 M NaCl, 10 mM Tris-HCl, 1 mM EDTA at pH=8, 0.5% SDS) with 1 μg of

proteinase K (Sigma-Aldrich) and incubated overnight at 55ºC. DNA was extracted using phenol/chloroform/isoamyl alcohol (PCI) after treatment with 0.5 µg of ribonuclease A (PureLink RNase A; Life Technologies). DNA was precipitated by 95% ethanol, eluted in Milli-Q® water (Merck, Millipore) and measured by ND-1000 spectrophotometer (NanoDrop Technologies).

MSAP: laboratory methods

The MSAP method was a modification of the protocol used by Morán and Pérez-Figueroa[1], in turn based on Reyna-López et al.[2] and Xu et al.[3]. Briefly, for each sample, digestion with restriction enzymes and ligation of adapters were performed simultaneously. For digestion, one aliquot of 100 ng of DNA was digested with 1 U of *Msp*I and 5 U of *Eco*RI in Multicore buffer (Promega) containing 1 µg of BSA, whereas a second aliquot of 100 ng of DNA was digested with 1 U of *Hpa*II and 5 U of *Eco*RI (Promega) in buffer A (Promega) also containing 1 µg of BSA. For ligation, 0.2 U of T4 ligase (Promega), 5 pmol of *Eco*RI adapter and 50 pmol of *Hpa*II/*Msp*I adapter (Table S4) were included in each digestion reaction. The mixtures were incubated at 37ºC for 2 h. Adapters were prepared by carefully mixing equal volumes of oligos, denaturing at 95ºC for 5 min and annealing at room temperature for 10 min. After 1:5 dilution, the produced fragments were amplified using pre-selective primers (Table S4) containing one additional nucleotide by 1 U of GoTaq® Flexi DNA Polymerase (Promega). PCR cycling parameters were as follows: 72ºC for 2 min, followed by 20 cycles of 94ºC for 20 s, 56ºC for 30 s and 72ºC for 2 min, and finally 30 min at 60ºC. Two combinations of selective primers were used to produce a sufficient number of bands for robust statistical scoring. *Hpa*II-*Msp*I-TC primers end-labeled using 6-FAM reporter dye were combined with *Eco*RI-ACT primers (Table S4). On the other hand, *Hpa*II-*Msp*I-TC primers end-labeled using HEX reporter dye were combined with *Eco*RI-AAG primers (Table S4). Selective amplifications were performed by 1 U of GoTaq® Flexi DNA Polymerase using touchdown cycling parameters: 94ºC for 5 min, followed by 12 cycles of 94ºC for 20 s, 0.7ºC decreasing in each cycle starting at 66ºC for 30s and 72ºC for 2 min, in turn followed by 23 cycles at 94ºC for 20 s, 56ºC for 30s and 72ºC for 2 min and finally 30 min at 60ºC. For each sample and pair of restriction enzymes, 1 µl of selective PCR was loaded with 15 µl of Hi-Di™ Formamide (Applied Biosystems) and 0.5 µl of GeneScan™ 500 ROX™ dye Size Standard (Applied Biosystems) on an ABI 3130xl or 3730xl Genetic Analyzer (Applied Biosystems).

MSAP: raw data processing

Peaks along the electropherograms were detected and sized by the Peak Scanner™ Software v1.0 (Applied Biosystems). All subsequent analyses were performed using R (v. 3.2.5) and Rstudio (v. 1.0.136)[4,5]. The definition of bins, i.e., the amplicon size categories, final filtering and further quality controls were performed with the R package RawGeno[6]. Bins with over 100 standard raw fluorescence units, inside the 50-

500 bp size range and reproducible over the 80% of replicated samples, were considered valid. Genotyping error per primer pair[7] was calculated by running two treatment groups, the 15ºC and 19ºC larvae (total of 35 samples), in duplicate and eight samples in triplicate from experiment 1 and seven samples in duplicate from experiment 2.

MSAP: classification of fragments according to methylation status

*Hpa*II and *Msp*I are isoschizomers that recognize the same nucleotide sequence (5'-CCGG-3') and have different sensitivity to the DNA methylation of the cytosines of the recognition sequence. Both enzymes cut unmethylated sequences and neither enzyme cuts hypermethylated sequences. In addition, *Msp*I cuts the 5'-C^{me}CGG-3'/3'-GG^{me}CC-5' and *Hpa*II cuts the 5'-^{me}CCGG-3'/3'-GGCC-5' sequence[8–12]. Therefore, bands were scored as follows: if they were present in both the *Hpa*II and *Msp*I digestions they were considered unmethylated (Type I); if they were present only in *Msp*I digestion they were considered methylated at the internal cytosine (Type II); if they were present only in the *Hpa*II digestion they were considered hemi-methylated in the outer cytosine (Type III); and if they were absent from both the *Hpa*II and *Msp*I digestions they were considered hypermethylated (Type IV). Regarding type IV loci, genetic variation was expected to be low and therefore the absence of restriction sites was more probable due to hypermethylation. Loci were classified as methylation-susceptible (MSL) or non-methylated (NML) if they were superior or inferior, respectively, to the Error Rate-based threshold (ERT). The ERT is specific for each primer combination, estimated as the proportion of discordant *Hpa*II-*Msp*I scores across individuals and indicative of scoring errors[13,14]. ERT was calculated by RawGeno as the mismatch error rate[7]. The error rate per primer was higher than in AFLP markers due to cell and tissue heterogeneity[14]. NML were too few to be considered as indicators of genetic variation and consequently they were eliminated from any further analysis. The binary matrices indicating band presence/absence for each enzyme per sample were transformed into binary matrices indicating methylated/unmethylated MSL per sample as follows: unmethylated (Type I) fragments were considered unmethylated, while hemi-methylated, methylated in the internal cytosine and hypermethylated fragments were considered methylated (Types II, III and IV). Due to the potential error in identifying hemi-methylated outer cytosines as *Hpa*II cuts[10], one additional metric of global methylation was calculated as the ratio: methylated loci (Types II and III)/scorable loci (Types I, II and III), as previously reported[15,16].

RNA extractions

Total RNA was extracted from 4 pools of ~10 whole larvae per group (experiment 1) and individually from upper body trunk of 4 juveniles per group (experiment 2) using the TRIzol reagent (Life Technologies) and precipitated by isopropyl alcohol. RNA quantity was measured by a ND-1000 spectrophotometer (NanoDrop Technologies) and

diluted to 200 ng/µl. Equilibrated RNA concentrations were measured by a ND-1000 spectrophotometer and normalized a second time.

Quantitative real-time PCR (qRT-PCR)

One microgram of RNA was treated with 0.5 U of DNase I (Life Technologies) and reverse transcribed to cDNA by the SuperScript III Reverse Transcriptase (Life Technologies) and 100 µM of random hexamers according to the manufacturer's instructions. New primers were designed using Primer3Plus[17]. Primers used for *nr3c1* were previously designed and validated in our group[18], while primers targeting reference genes, the elongation factor-1 alpha (*ef-1a*) and the 40S ribosomal protein S30 (*fau*), had been previously validated in sea bass[19]. These two reference genes were used for normalization after evaluating also the ribosomal protein L13a (*L13a*)[19] and the ribosomal protein S18 (*S18*) and were chosen because of their stable expression among groups and an amplification efficiency close to 2. Primers sequences are shown in Table S5.

All primers were validated separately in larvae and juveniles by performing serial dilutions (1, 1:5, 1:10, 1:50, 1:100, 1:500) of one pool of 5 cDNA samples from the 17ºC group and 5 cDNA samples from 15-19 (240) group and a second pool of 5 cDNA samples from the 17ºC group and 5 cDNA samples from the 21ºC group from experiment 2. These dilutions were used to perform calibration curves in order to calculate the slopes of log-linear regressions, correlation coefficients (r^2) and amplification efficiencies as $E=10^{(-1/slope)}$ for each primer pair (Table S5). The specificity of the qPCR was confirmed by melting curve analysis (95ºC, 60ºC and 95ºC each for 15 s). SYBR Green (Life Technologies) chemistry was used in qRT-PCR. Reactions were performed in a total volume of 10 µl using 0.5 µl of each primer at 10 µM concentration, 5 µl of SYBR Green and 2 µl of cDNA, where the maximum dilution that produced Cq values <30 was used for each primer (Cq is used instead of Ct following MIQE guidelines). Reactions were run in triplicate and negative controls lacking cDNA were included in duplicate. qRT-PCR reactions were carried out on an ABI 7900HT (Applied Biosystems) unit with the following cycling parameters: UDG decontamination at 50ºC for 2 min, initial activation at 95ºC for 10 min and 40 cycles of denaturation at 95ºC for 15 s and annealing/extension at 60ºC for 1 min. A dissociation step (95ºC, 60ºC and 95ºC each for 15 s) was added at the end of each run.

Data availability

All data generated and analysed during this study are included as Supplementary Information files. These include the binary files used as inputs for the msap program, those with the methylation-sensitive polymorphic loci (outputs of the msap) on which the DNA methylation statistics were based and the dCq values of RT-qPCR.

Supplementary Figures

Figure S1. Schematic representation of the temperature treatments during development. The thermosensitive period in the European sea bass expands from 0 to ~60 days post fertilization (dpf). In experiment 1, fish were either reared at constant 15°C, 17°C or 19°C, or they were exposed to temperature changes at 15, 120 or 240 hours post fertilization (hpf) after an acclimation either at 15°C or 19°C. The treatments lasted from 0 to 15 dpf when all fish were sampled as larvae. The whole experiment was repeated 5 times. In experiment 2, natural fluctuations of temperature (around 17°C) occurred until 20 dpf. Then one group of larvae was subjected to constant 17°C or constant 21°C until 60 dpf when all fish were sampled as juveniles. Each group was carried out in duplicate.

Figure S2. Experimental set-up and temperature treatments of experiment 1. Eggs from 5 different spawns coming from different broodstock kept in 5000-liter tanks were used. The eggs and larvae of each spawn were kept separate. Each batch of eggs was split in between three and five 19-liter cylindrical containers, depending on batch size, and first placed inside 650-liter fiberglass tanks at 14°C. There were 10 additional 650-liter tanks for the temperature experiments. Constant temperature experiments involved three groups reared at constant 15°C, 17°C, or 19°C. Six additional groups were exposed to temperature changes after acclimation at either 15°C or 19°C. Temperature changes took place at 15, 120 or 240 hours post fertilization (hpf) and all fish were sampled at 15 days post fertilization. Within a tank, temperature fluctuations were always <1°C. Pseudoreplication was avoided by using different families and replicate 19-liter cylinders for each family. Tank effects were avoided by having each temperature in duplicate and because the 19-liter tanks were moved across several 650-liter tanks to achieve the desired temperature.

Figure S3. Overview of raw Methylation Sensitive Amplification Polymorphism data. (a-b) Agarose gel electrophoresis from selective amplifications of a subset of samples. The subset of samples includes genomic DNA from pools of larvae reared at constant 17ºC, switched from 15ºC to 19ºC and from 19ºC to 15ºC at 120 hours post fertilization (hpf). For each sample, two separate double enzyme digestions were produced: with *Msp*I/*Eco*RI and with *Hpa*II/*Eco*RI. After a pre-selective PCR step, for each digestion two selective PCRs were performed with primers that included three selective nucleotides (ACT or AAG). At the same time, the second primer of the PCR was labeled with HEX or 6-FAM and included two selective nucleotides (TC). For samples 1, 2 … 19, *Hpa*II/*Eco*RI digestions are shown after selective amplification with the AAG selective primer and HEX labeled (a), and with the ACT selective primer and 6-FAM labeled (b). In each case, in the first well the TrackIt 100 bp DNA Ladder (Life Technologies) was loaded, and in the last well, a negative control (NC) ran through the entire MSAP protocol without genomic DNA was loaded to the 2% agarose gels. Five

microliters of the 20 µl total selective PCR reaction volumes were loaded for each sample. (c-d) Electropherograms after fluorescence-based capillary electrophoresis were visualized by Peak Scanner™ Software v1.0 (Applied Biosystems). For one sample (sample 2.2) of the 17ºC larvae group, one *Msp*I/*Eco*RI (c) and one *Hpa*II/*Eco*RI (d) digestions are shown after selective amplification with the ACT selective primer and 6-FAM-labeled (blue), and with the AAG selective primer and HEX- labeled (green). In all cases, the MSAP protocol was initiated with 100 ng of genomic DNA.

Figure S4. Frequencies of methylation-susceptible polymorphic loci (MSL) band types. Shown are the percent of bands corresponding to unmethylated (Type I), methylated in the inner cytosine (Type II), hemi-methylated in the outer cytosine (Type III) and the ratio of Type II+Type III over the scorable loci (Type I+Type II+Type III) in the 15 days post-fertilization (dpf) larvae reared at 15ºC (15ºC-15 dpf) or at 19ºC (19ºC-15 dpf) or in the juveniles at 60 dpf reared at 17ºC (17ºC-60 dpf) or at 21ºC (21ºC-60 dpf). The sample sizes were as follows: Larvae: 15ºC, n=21, 19ºC, n=12; Juveniles: 17ºC, n=18, 21ºC, n=18. Fully methylated MSL are not included.

Figure S1

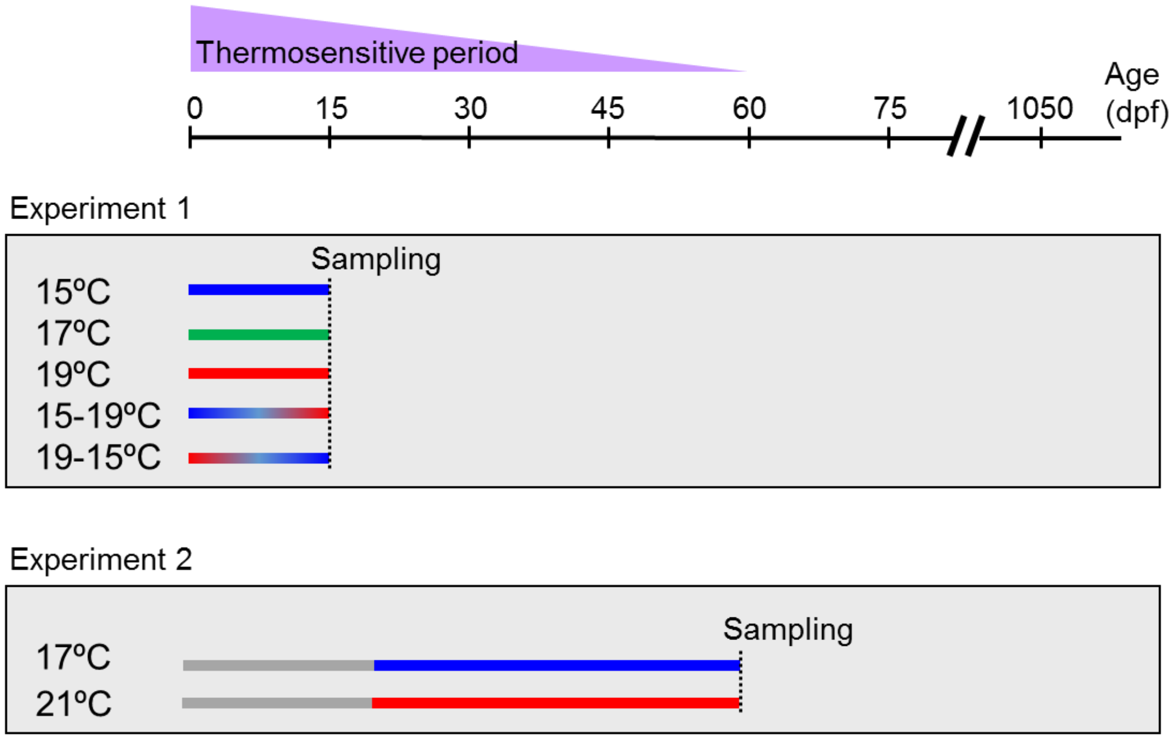
Thermosensitive period
0
15
30
45
60
75
1050
Age (dpf)
Experiment 1
Sampling
15ºC
17ºC
19ºC
15-19ºC
19-15ºC
Experiment 2
Sampling
17ºC
21ºC

Figure S2

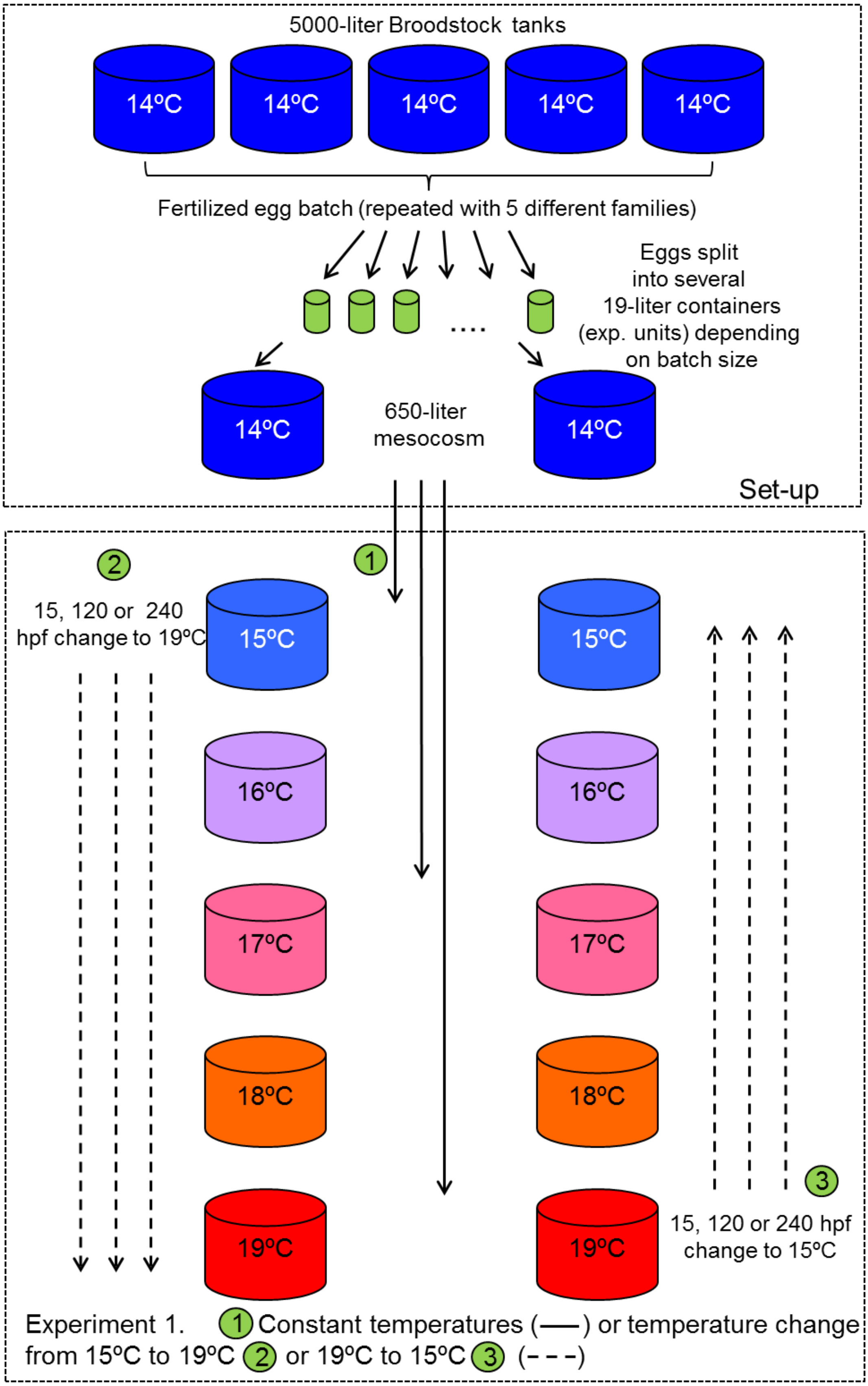
5000-liter Broodstock tanks
14°C
14°C
14°C
14°C
14°C
Fertilized egg batch (repeated with 5 different families)
Eggs split into several 19-liter containers (exp. units) depending on batch size
....
14°C
650-liter mesocosm
14°C
Set-up
2
15, 120 or 240 hpf change to 19°C
1
15°C
15°C
16°C
16°C
17°C
17°C
18°C
18°C
19°C
19°C
3
15, 120 or 240 hpf change to 15°C
Experiment 1. 1 Constant temperatures (——) or temperature change from 15°C to 19°C 2 or 19°C to 15°C 3 (- - -)

Figure S3

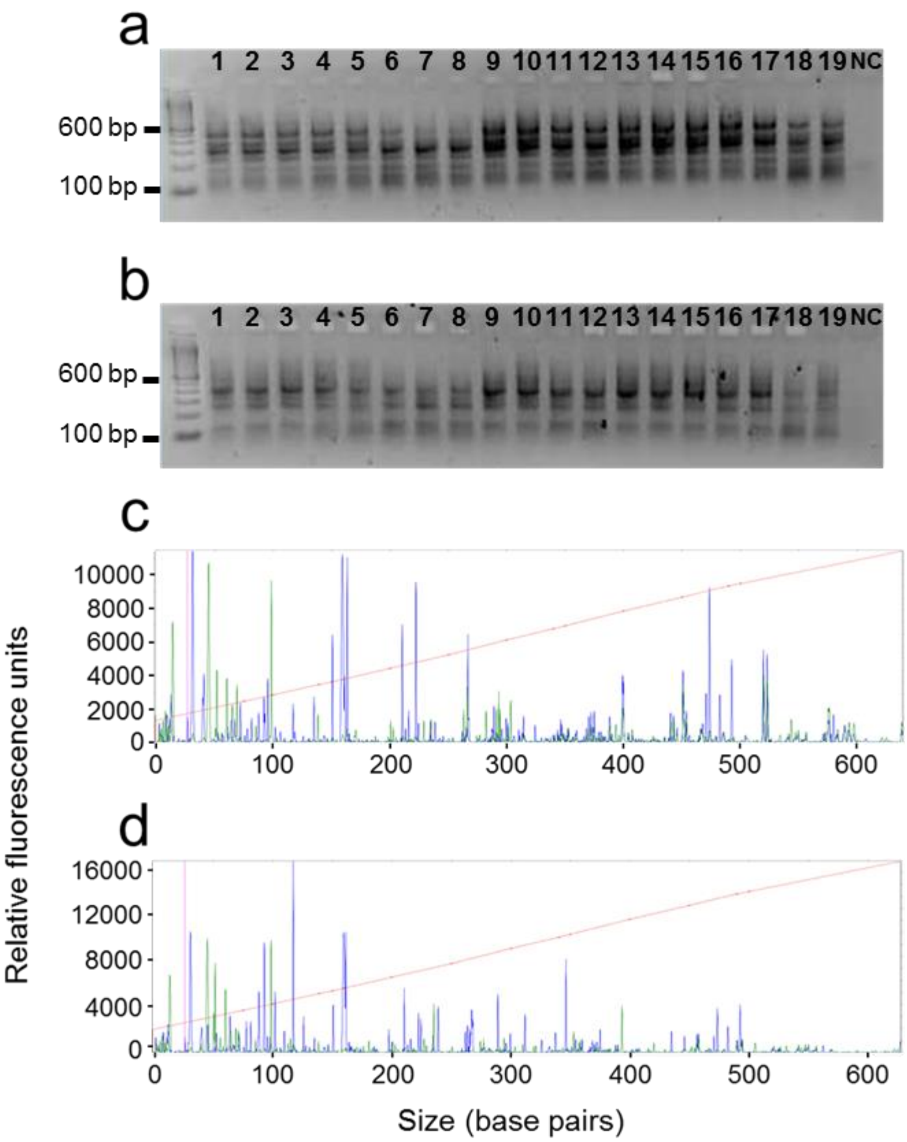
a
1 2 3 4 5 6 7 8 9 10 11 12 13 14 15 16 17 18 19 NC
600 bp
100 bp
b
1 2 3 4 5 6 7 8 9 10 11 12 13 14 15 16 17 18 19 NC
600 bp
100 bp
c
10000
8000
6000
4000
2000
0
0 100 200 300 400 500 600
d
16000
12000
8000
4000
0
0 100 200 300 400 500 600
Relative fluorescence units
Size (base pairs)

Figure S4

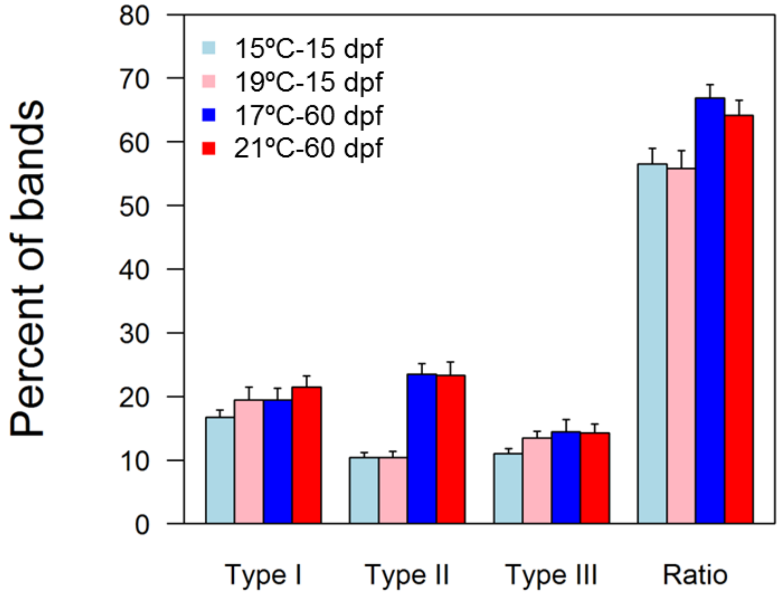

Percent of bands
80
70
60
50
40
30
20
10
0
15ºC-15 dpf
19ºC-15 dpf
17ºC-60 dpf
21ºC-60 dpf
Type I
Type II
Type III
Ratio

Supplementary Tables

Table S1. Summary of the experimental treatments carried out in this study. The appellation of experiments and groups is indicated with the corresponding temperature, period of temperature treatment and sampling point

Experiment	Group	Temperature (ºC)	Period (dpf)	Sampling (dpf)
1.1	15	15	0-15	15
1.1	17	17	0-15	15
1.1	19	19	0-15	15
1.2	15-19(15)	15-19	0-15	15
1.2	15-19(120)	15-19	0-15	15
1.2	15-19(240)	15-19	0-15	15
1.2	19-15(15)	19-15	0-15	15
1.2	19-15(120)	19-15	0-15	15
1.2	19-15(240)	19-15	0-15	15
2	17	17	20-60	60
2	21	21	20-60	60

Abbreviations: dpf, days post fertilization.

Table S2. MSAP loci for each comparison of 0-15 days post fertilization (dpf) and for 20-60 dpf fish. The mismatch error rate is indicated for selective primer 1 (SP1; Sel_ECORI+ACT) and selective primer 2 (SP2; Sel_ECORI+AAG). The number of loci, the number of methylation-susceptible loci (MSL) and nonmethylated loci (NML) are indicated for each primer and in total, while the total number of polymorphic MSL, the percentage of loci which are polymorphic MSL and the total number of polymorphic NML are also indicated

Period (dpf)	Treatment (ºC)		Error rate	Loci	MSL	NML	Polymoprhic MSL	Polymoprhic NML
0-15	15 *vs*. 17 *vs*. 19	SP1	0.2	252	223	29		
		SP2	0.18	241	207	34		
		Total		493	430	63	298 (60.45%)	62
	15→19	SP1	0.12	214	200	14		
		SP2	0.09	201	199	2		
		Total		415	399	16	231 (55.66%)	16
	19→15	SP1	0.12	214	198	16		
		SP2	0.09	201	196	5		
		Total		415	394	21	204 (49.16%)	21
20-60	17 *vs*. 21	SP1	0.35	174	151	23		
		SP2	0.09	164	156	8		
		Total		338	307	31	216 (63.91%)	31

Table S3. Pairwise Φ_{ST} between larvae subjected to constant temperature from 0 to 15 days post fertilization (Experiment 1.1)

Temperature (°C)	Φ_{ST}	*p*-value
15 *vs*. 17	0.2149	<0.0001
15 *vs*. 19	0.0759	0.0064
17 *vs*. 19	0.1822	<0.0001

Table S4. Oligos used in MSAP

Function	Name	Sequence	Labeling
Adapters	Ada_ECORI_R	AATTGGTACGCAGTCTAC	
	Ada_ECORI_F	CTCGTAGACTGCGTACC	
	Ada_HPA/MSP_R	GACGATGAGTCTAGAA	
	Ada_HPA/MSP_F	CGTTCTAGACTCATC	
Pre-selective primers	Pre_ECORI+A	GACTGCGTACCAATTCA	
	Pre_HPA/MSP+T	GATGAGTCTAGAACGGT	
Selective primers	Sel_ECORI+ACT	GACTGCGTACCAATTCACT	
	Sel_ECORI+AAG	GACTGCGTACCAATTCAAG	
	Sel_HPA/MSP+TC	GATGAGTCTAGAACGGTC	HEX
	Sel_HPA/MSP+TC	GATGAGTCTAGAACGGTC	6-FAM

Table S5. Characteristics of RT-qPCR primers and amplicons

Gene	Gene symbol	Primer sequence (5'→3')	Efficiency (*E*)	Slope	r^2	Tm	Product size (bp)	Genomic Position
Epigenetic regulation								
DNA (cytosine-5-)-methyltransferase 1	*dnmt1*	(F)AAAGGCGTTGCTGGGAAAAG<br>(R)TGGTCACTTCTTCACTGTCCTC	2.04	-3.22	0.99	59.6<br>59.6	125	LG8:11025828-11037878
DNA (cytosine-5-)-methyltransferase 3	*dnmt3*	(F)ACTGTCTGAACATCCTCATCGG<br>(R)ATTCCTGCACACGAATGCTC	2.15	-3.01	0.98	59.8<br>58.9	141	LG22-25:1883800-1900186
Growth								
Insulin-like growth factor 1	*igf1*	(F)CACACAGACATGCCAAGAGC<br>(R)TTTGTCTTGTCTGGCTGCTG	1.99	-3.34	0.98	59.5<br>58.7	98	LGx:15009651-15022355
Thyroid metabolism								
Thyroid hormone receptor alpha	*tr-α*	(F)AATGGGAGCTGATCAGGATGG<br>(R)ACTTGCGCTTCTGTTTCCAG	2.02	-3.29	0.93	59.6<br>59.1	81	LG8:21012807-21041447
Muscle								
Myogenin	*myog*	(F)TGACAGGTACAGAGGACAAAGC<br>(R)ACGGTCCATGGTAACTGTCTTC	2.27	-2.81	0.97	60.0<br>60.0	125	LG1A:13290583-13292192
Vision								
Long melanopsin	*opn4a*	(F)TGGATCTGTCTGTGCGTCAC<br>(R)ACGCAGCACTTCTGTTGTTC	2.17	-2.97	0.99	60.0<br>59.3	83	LG11:23540693-23550809
Digestive system								
Trypsinogen 2	*tryp2*	(F)CTTGGTGAGCACAACATTGC<br>(R)ATGTCATTGTCCAGGTTGCG	2.20	-2.92	0.99	58.2<br>58.8	107	LG16:12858222-12859429
Heat shock response								
Heat shock cognate 70	*hsp70*	(F)TCACTAAGCTGTACCAGAGTGC<br>(R)AATCGACCTCCTCAATGGTTGG	2.08	-3.14	1.00	59.8<br>60.4	135	LG14:4354065-4355815

Stress								
Glucocorticoid receptor	*nr3c1*	(F)CTTCCATCCAGCCCGTTGAT<br>(R)GTAGTGGAGGTCTGCGTCTG	2.15	-3.01	0.99	60.1<br>59.8	185	LG2:10375856-10392681
Reference genes								
Elongation factor-1 alpha	*ef-1a*	(F)AAATGCGGAGGAATCGACAA<br>(R)GAGCCCTTGCCCATCTCAG	2.07	-3.16	0.98	62.4<br>60.2	71	LG9:21150983-21153402
40S ribosomal protein S30	*fau*	(F)GACACCCAAGGTTGACAAGCAG<br>(R)GGCATTGAAGCACTTAGGAGTTG	2.04	-3.24	0.99	61.9<br>60.1	149	LG17:7487904-7489704

Supplementary References

1. Morán, P. & Pérez-Figueroa, A. Methylation changes associated with early maturation stages in the Atlantic salmon. *BMC Genet.* **12,** 86 (2011).
2. Reyna-López, G. E., Simpson, J. & Ruiz-Herrera, J. Differences in DNA methylation patterns are detectable during the dimorphic transition of fungi by amplification of restriction polymorphisms. *Mol. Gen. Genet. MGG* **253,** 703–710 (1997).
3. Xu, M., Li, X. & Korban, S. S. AFLP-based detection of DNA methylation. *Plant Mol. Biol. Report.* **18,** 361–368 (2000).
4. R Core Team. *R: A Language and Environment for Statistical Computing*. (R Foundation for Statistical Computing, 2015).
5. RStudio Team. *RStudio: Integrated Development Environment for R*. (RStudio, Inc., 2015).
6. Arrigo, N., Tuszynski, J. W., Ehrich, D., Gerdes, T. & Alvarez, N. Evaluating the impact of scoring parameters on the structure of intra-specific genetic variation using RawGeno, an R package for automating AFLP scoring. *BMC Bioinformatics* **10,** 33 (2009).
7. Bonin, A. *et al.* How to track and assess genotyping errors in population genetics studies. *Mol. Ecol.* **13,** 3261–3273 (2004).
8. Wenzel, M. A. & Piertney, S. B. Fine-scale population epigenetic structure in relation to gastrointestinal parasite load in red grouse (*Lagopus lagopus scotica*). *Mol. Ecol.* **23,** 4256–4273 (2014).
9. Liu, S. *et al.* Natural epigenetic variation in the female great roundleaf bat (*Hipposideros armiger*) populations. *Mol. Genet. Genomics* **287,** 643–650 (2012).
10. Fulneček, J. & Kovařík, A. How to interpret methylation sensitive amplified polymorphism (MSAP) profiles? *BMC Genet.* **15,** 2 (2014).
11. Yang, C. *et al.* Analysis of DNA Methylation in Various Swine Tissues. *PLoS ONE* **6,** e16229 (2011).

12. Schulz, B., Eckstein, R. L. & Durka, W. Scoring and analysis of methylation-sensitive amplification polymorphisms for epigenetic population studies. *Mol. Ecol. Resour.* **13,** 642–653 (2013).
13. Herrera, C. M. & Bazaga, P. Epigenetic differentiation and relationship to adaptive genetic divergence in discrete populations of the violet *Viola cazorlensis*. *New Phytol.* **187,** 867–876 (2010).
14. Herrera, C. M. & Bazaga, P. Epigenetic correlates of plant phenotypic plasticity: DNA methylation differs between prickly and nonprickly leaves in heterophyllous *Ilex aquifolium* (Aquifoliaceae) trees. *Bot. J. Linn. Soc.* **171,** 441–452 (2012).
15. Nicotra, A. B. *et al.* Adaptive plasticity and epigenetic variation in response to warming in an Alpine plant. *Ecol. Evol.* **5,** 634–647 (2015).
16. Ardura, A., Zaiko, A., Morán, P., Planes, S. & Garcia-Vazquez, E. Epigenetic signatures of invasive status in populations of marine invertebrates. *Sci. Rep.* **7,** 42193 (2017).
17. Untergasser, A. *et al.* Primer3—new capabilities and interfaces. *Nucleic Acids Res.* **40,** e115 (2012).
18. Díaz, N. & Piferrer, F. Lasting effects of early exposure to temperature on the gonadal transcriptome at the time of sex differentiation in the European sea bass, a fish with mixed genetic and environmental sex determination. *BMC Genomics* **16,** 679 (2015).
19. Mitter, K. *et al.* Evaluation of candidate reference genes for QPCR during ontogenesis and of immune-relevant tissues of European seabass (*Dicentrarchus labrax*). *Comp. Biochem. Physiol. B Biochem. Mol. Biol.* **153,** 340–347 (2009).
